# Unveiling secrets of traditional Chinese medicine: Cutting-edge techniques in component analysis

**DOI:** 10.1016/j.chmed.2025.05.006

**Published:** 2025-05-23

**Authors:** Tingting Zhou

**Affiliations:** Department of Pharmaceutical Analysis, School of Pharmacy, Second Military Medical University, Shanghai 200433, China; Shanghai Key Laboratory for Pharmaceutical Metabolite Research, School of Pharmacy, Second Military Medical University, Shanghai 200433, China

**Keywords:** data processing, instrumental technology, online activity analysis, pretreatment methods, TCM component analysis

## Abstract

Chemical component analysis is a critical challenge in Chinese herbal medicine research, involving the qualitative and quantitative identification of complex constituents in traditional Chinese medicine (TCM). However, traditional analytical methods are insufficient for efficient and comprehensive analysis of complex composition of TCM. Limitations exist in sample preparation, instrumental technology, data processing, and activity-related quality marker research. Recent advancements have significantly improved analytical precision, enabling more comprehensive profiling of TCM components. New pretreatment methods improve extraction efficiency and detection sensitivity, while novel instrumental technologies, such as mass spectrometry imaging, preserve spatial information lost in homogenization. AI enhances data interpretation, improving accuracy and efficiency. Online activity analysis links chemical composition with bioactivity, overcoming the limitations of purely chemical profiling and enabling a more comprehensive evaluation of TCM efficacy. This perspective provides an overview of the development trends in component analysis, aiming to advance the field and support TCM modernization.

Traditional Chinese medicine (TCM), as a fundamental component of traditional medical systems, is characterized by its multi-component, multi-target, and multi-pathway therapeutic features, which stem from its complex chemical composition and diverse biological activities. This intrinsic complexity poses significant challenges for component analysis, underscoring the necessity of comprehensive analytical approaches. A thorough analysis of TCM not only helps to elucidate its material basis and mechanisms of action but also provides scientific support for efficacy tracing, identification of active regions, and quality consistency assurance. Moreover, it plays a pivotal role in the improvement of quality standards, product uniformity, and clinical efficacy evaluation. As the paradigm of evidence-based medicine continues to evolve, comprehensive analysis of TCM is becoming increasingly indispensable in advancing the modernization of traditional medicine and supporting its scientific application in clinical settings.

Despite the significant importance of comprehensive analysis in TCM, numerous technical bottlenecks persist, necessitating urgent breakthroughs and innovations. Firstly, the complex chemical composition and broad polarity range of TCM compounds complicate traditional separation methods, which often fail to simultaneously capture both highly polar and non-polar compounds, leading to incomplete information. Therefore, optimizing pretreatment techniques is crucial to enhance extraction efficiency and expand component coverage. Secondly, homogenization during sample preparation results in the loss of spatial information, whereas *in situ* analytical techniques preserve the spatial distribution characteristics of TCM components, facilitating the identification of pharmacologically active regions. Thirdly, TCM analysis involves vast and complex datasets, yet conventional analytical approaches face limitations in high-throughput and efficient screening. Thus, advanced data processing technologies are essential for rapid and precise component identification and quantification, enhancing analytical efficiency. Finally, traditional methods primarily focus on detecting chemical constituents while overlooking their biological activity. Activity-oriented analytical techniques establish a direct correlation between chemical composition and pharmacological effects, providing essential support for a comprehensive evaluation of TCM efficacy. In recent years, continuous advancements in pretreatment optimization, the development of novel instrumental technologies, and the integration of artificial intelligence-based data processing with online activity analysis have significantly expanded both the depth and breadth of component analysis in TCM. These technological advancements have not only improved the accuracy and efficiency of qualitative and quantitative analyses but also enhanced the spatial and temporal resolution, enabling a clearer and more intuitive understanding of the correlations between chemical components and biological activities. These breakthroughs have provided new momentum for research on the quality of TCM and its clinical applications, thereby accelerating the scientific, modernized, and international development of TCM. This perspective elucidates the application of these cutting-edge technologies in the analysis of complex TCM systems and anticipates their future potential in advancing research (Fig. 1).

**Fig. 1:**
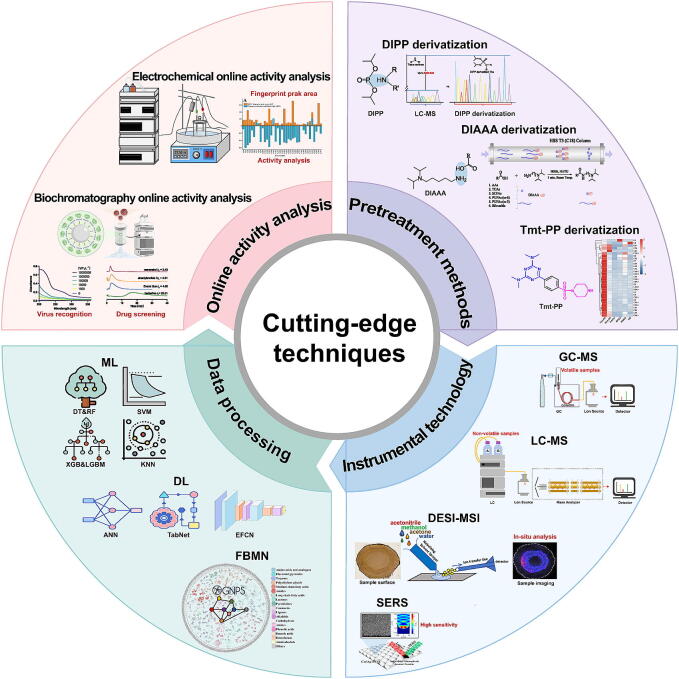
Cutting-edge techniques in component analysis.

## 1 Pretreatment methods

Efficient sample preparation is essential for accurately analyzing the complex constituents of TCM. Typical preprocessing steps include extraction, concentration and drying, purification and separation, and derivatization. Conventional separation methods often show bias toward specific TCM components, particularly when dealing with compounds that have significant polarity differences, such as peptides and fatty acids, where conventional techniques often struggle to achieve effective separation. This limitation hinders the comprehensive characterization of diverse chemical profile of TCM. Recent advancements in derivatization methods have been developed to overcome this challenge. These methods involve chemical modifications that increase the hydrophilicity of low-polarity compounds or enhance the hydrophobicity of highly polar ones, thereby improving their separation properties and significantly enhancing resolution. Compared with conventional methods, derivatization not only improves separation efficiency but also enhances analytical sensitivity and accuracy by reducing matrix interference and increasing ionization efficiency, providing critical support for the comprehensive analysis of TCM components (Xia & Wan, 2023). Several newly developed derivatization methods further optimize the analysis of TCM’s complex chemical components. For example, (Diisopropylamino)pentylamine (DIAAA) improves separation by modifying hydroxyl-containing metabolites while 5-diisopropylphosphite (DIPP) selectively enhances the ionization efficiency of amine-containing compounds (Gao et al., 2023, Bian et al., 2023). Additionally, microwave-assisted Tmt-PP (*p*-[3,5-(dimethylamino)-2,4,6-triazine] benzene-1-sulfonyl piperazine) derivatization significantly reduces reaction time while enhancing the stability of derivatized products, addressing the inefficiency of conventional derivatization methods (Tang et al., 2021). While derivatization introduces some complexity to sample preparation, it effectively minimizes matrix interference, mitigates the negative impact of increased moisture content on ionization efficiency, and significantly enhances detection sensitivity and resolution. Notably, these advancements have facilitated the investigation of highly water-soluble primary metabolites, which were previously difficult to analyze using traditional separation techniques. As a result, derivatization is widely applied in TCM analysis, greatly improving the comprehensiveness and accuracy of complex component profiling.

## 2 Instrumental technology

One of the difficulties in TCM component analysis is the challenge of achieving a comprehensive and accurate profiling of its compounds. A key advancement in TCM analysis is the development of novel analytical instruments, enhancing both sensitivity and precision. Currently, most research utilizes advanced instrumental technologies, including chromatography, mass spectrometry (MS), and spectroscopy, along with their hyphenated forms, to separate and identify the complex components of TCM (Li et al., 2024). Gas chromatography (GC) enables fast and sensitive analysis of volatile compounds, while liquid chromatography (LC) offers good separation but requires extensive preparation and prolonged time. However, conventional chromatographic techniques often struggle with the simultaneous separation of compounds with extreme polarity differences, leading to biased detection of certain constituents. Recent advancements have focused on optimizing chromatographic conditions and developing new stationary phases to address this issue. Mass spectrometry and its coupled techniques, such as GC/MS and LC/MS, enable accurate qualitative and quantitative analysis of complex TCM samples. Despite their high sensitivity, conventional MS techniques are often affected by matrix effects, thus reducing analytical accuracy. To address this limitation, recent research has explored optimized ionization techniques and advanced data acquisition strategies to minimize matrix interference and improve detection sensitivity. Herein, imaging techniques such as matrix-assisted laser desorption/ionization mass spectrometry imaging (MALDI-MSI) and desorption electrospray ionization mass spectrometry imaging (DESI-MSI) have been developed. These techniques allow for the *in situ* observation of the spatial distribution of drug components, providing valuable insights into the localization of TCM constituents. Compared to MALDI-MSI, DESI-MSI does not require a matrix coating, thus avoiding issues related to uneven signals and excessive noise peaks (Gao et al., 2025). However, conventional DESI-MSI has limitations in sensitivity for detecting low-abundance analytes and relatively lower spatial resolution. Recent improvements have focused on refining ionization efficiency, enhancing spatial resolution, and integrating multi-modal imaging techniques to provide a more comprehensive visualization of chemical distributions. Spectroscopic methods, including infrared spectroscopy (IR), ultraviolet–visible spectroscopy (UV–Vis), nuclear magnetic resonance spectroscopy (NMR), surface-enhanced Raman spectroscopy (SERS), and fluorescence spectroscopy, are among the primary methods for qualitative analysis (Liu et al., 2025). These methods do not require pure samples, offer high selectivity and sensitivity, and cause minimal sample damage. Additionally, they allow for simultaneous detection of multiple elements or compounds, eliminating the need for complex separation procedures, but challenges remain in handling spectral interference and extracting meaningful information from large, complex datasets. The integration of AI-driven spectral processing techniques has been explored to enhance signal deconvolution, reduce spectral noise, and improve analytical accuracy. These advanced techniques have significantly improved the precision and depth of TCM analysis. By integrating these technological advancements, researchers can overcome the limitations of traditional methods, achieving a more comprehensive, high-throughput, and accurate analysis of TCM components. It is necessary to integrate these technical advantages, leveraging each method’s strengths according to the component characteristics of different complex molecular systems in TCM.

## 3 Data processing

The complexity of TCM formulations requires sophisticated data processing techniques to interpret the vast amounts of data generated by modern analytical instruments. Traditional analysis has largely relied on researchers’ expertise for qualitative analysis, which is time-consuming, subjective, and inefficient for handling large and complex datasets. Additionally, conventional data processing methods struggle with extracting meaningful information from noisy, multi-dimensional data, limiting the depth of chemical characterization in TCM research. With advances in computational chemistry and the integration of artificial intelligence (AI) technologies, data processing has evolved from manual interpretation to automated, high-throughput analysis, significantly improving efficiency and accuracy. These methods effectively extract characteristic chemical information from vast datasets, streamline analysis workflows, and deliver precise results. This overcomes the limitations of manual analysis and elevates the intelligence level of TCM component analysis. Currently, novel computational chemistry methods applied to TCM analysis include AI and feature-based molecular networking (FBMN), both of which provide innovative solutions to overcome the challenges of conventional data interpretation. Machine learning (ML) and deep learning (DL) are crucial components of AI. While ML utilizes interpretable algorithms for data pattern recognition, it faces limitations in handling high-dimensional, multimodal data and requires extensive feature engineering. In contrast, DL addresses these issues by employing multilayer nonlinear mappings, allowing for more effective feature extraction and pattern recognition in complex TCM datasets. AI is based on detailed statistical principles and robust computational power, provides an ideal platform for analyzing complex data from techniques like SERS and MS, and for advancing multi-omics research in TCM (Liu et al., 2023, Zhang et al., 2024). Moreover, molecular networking has transformed mass spectrometry data processing and visualization. It is a data visualization tool that constructs a mass spectrometry data network based on the structural similarity of detected compounds to assist in qualitative analysis. Compared with traditional MS data processing methods, rely on manual spectral matching, FBMN automates the clustering and annotation of related compounds, significantly improving the speed and accuracy of compound identification. FBMN rapidly clusters and visualizes extensive mass spectrometry feature data by integrating multiple variables, such as ion mobility and isotopic patterns, thereby enhancing structural elucidation and metabolite annotation in TCM research. The application of FBMN and other technologies in TCM research not only accelerates the discovery of new compounds but also facilitates a deeper understanding of the chemical diversity, metabolic pathways, and potential synergistic effects within TCM formulations, overcoming the limitations of conventional qualitative analysis (Gao et al., 2023).

## 4 Online activity analysis

The complexity of TCM components presents significant challenges in screening bioactive compounds. Activity-guided analysis of the chemical components in TCM is an effective method for identifying active ingredients. Bioassays are conducted during the separation process to screen for bioactive compounds, followed by the targeted purification of these active ingredients. Therefore, the use of activity-guided screening for quality markers greatly enhances the separation efficiency of active components and provides a more precise understanding of the intrinsic relationship between the quality of TCM and its therapeutic efficacy, thereby offering a solid foundation for the scientific evaluation of TCM quality and effectiveness. Online activity analysis techniques offer a promising strategy for screening bioactive compounds. Compared with traditional bioassays, online activity analysis require offline fractionation and time-consuming activity testing, which enables real-time screening, significantly improving efficiency and reducing sample loss. Current online activity analysis methods primarily utilize electrochemical techniques or biochromatography to screen and monitor active components in real-time. These methods are often coupled with separation techniques, allowing for simultaneous separation and activity assessment of sample components. Electrochemical methods generally offer high sensitivity, enabling the detection of low concentrations of antioxidant chemical components and often providing results in a short time with minimal sample volume. However, electrochemical reactions are sensitive to the electrode materials used, which could lead to varying electrochemical behaviors depending on the materials. Additionally, the results may be influenced by environmental factors such as the pH of the solution, temperature, and electrolyte concentration. Certain antioxidants may not produce detectable electrochemical signals, which could represent a future direction for the development of electrochemical fingerprinting (Yang et al., 2024). Biological chromatography typically utilizes proteins, receptors, or DNA as stationary phases or in tandem stationary phases to separate active molecules in TCM based on their binding affinities. Among these techniques, critical micelle concentration (CMC) employs cell membranes as the stationary phase, simulating the physiological environment *in vivo*. Due to its biocompatibility, CMC offers high specificity and sensitivity, making it particularly well-suited for the activity screening of complex samples. With innovations in membrane materials and ongoing technological advancements, CMC is expected to achieve breakthroughs in improving separation efficiency, enhancing result reproducibility, and expanding its application scope. Additionally, the integration of CMC with mass spectrometry (CMC-MS) has enabled direct identification of active components, reducing the need for extensive purification steps and accelerating the discovery of bioactive compounds. These developments will offer more precise and efficient solutions for the screening of bioactive components in TCM (Ge et al., 2023). In conclusion, online activity analysis holds promising potential for broad applications. By addressing the limitations of traditional screening methods, these advancements enable a more comprehensive and high-throughput approach to identifying bioactive compounds in complex TCM systems. With rapid advancements in these technologies, integrating complex molecular systems with online activity analysis is expected to effectively address the challenges mentioned above.

## 5 Conclusion

This perspective highlights recent advancements in sample pretreatment, instrumental technology, data processing, and online activity analysis, which are transforming the landscape of TCM component analysis. By integrating cutting-edge technologies, these advancements not only improve the precision and efficiency of identifying complex TCM constituents but also pave the way for the discovery of novel bioactive compounds and the exploration of their mechanisms of action. The evolution of these analytical techniques is crucial for unraveling the complex molecular landscape of TCM, ultimately supporting its modernization, global integration, and wider acceptance in the biomedical field. These innovations have the potential to bridge traditional practices with contemporary scientific paradigms, ensuring that TCM remains a relevant and valuable resource in modern healthcare.

## Declaration of competing interest

The authors declare that they have no known competing financial interests or personal relationships that could have appeared to influence the work reported in this paper.
